# Assessing the relationships between capability, opportunity, and motivation in influencing self-isolation behaviour during pandemics

**DOI:** 10.1038/s41598-026-36198-7

**Published:** 2026-01-14

**Authors:** Gbeminiyi J. Oyedele, Ankit Shanker, Michael J. Tildesley, Ivo Vlaev

**Affiliations:** 1 Institute for Global and Pandemic Planning, University of Warwick, CV4 7AL Coventry, UK; 2 Warwick Medical School, University of Warwick, CV4 7AL Coventry, UK; 3 Centre for Behavioural and Implementation Science Intervention (BISI), Yong Loo Lin School of Medicine, National University of Singapore, 117597 Singapore, Singapore; 4 School of Life science and Mathematics, University of Warwick, CV4 7AL Coventry, UK

**Keywords:** COM-B, Self-isolation, Coronavirus disease, Behavioural change, Health care, Psychology, Psychology

## Abstract

Adherence to self-isolation was a central measure for controlling the spread of COVID-19; however, compliance varied widely. Understanding the behavioural determinants that drive adherence is critical for informing future public health intervention. This study applied the COM-B model to examine the associations between capability, opportunity, motivation, and self-isolation behaviour during the COVID-19 pandemic in the United Kingdom. A retrospective analysis was conducted using secondary data from the UK Office for National Statistics 2019 Opinions and Lifestyle Survey, which was not originally designed to measure COM-B constructs. Structural equation modelling (SEM) was used to examine the relationships between capability, opportunity, motivation, and self-isolation behaviour. Opportunity and motivation were significantly associated with self-isolation, while capability was linked to behaviour indirectly through its association with motivation, reflecting a possible pathway suggested by the structural model. Although some measurement indicators demonstrated lower reliability owing to the use of secondary data, the overall model fit was good (RMSEA = 0.049, CFI = 0.966, TLI = 0.944, SRMR = 0.040). These findings highlight the dominant influence of social and motivational factors in shaping adherence. This study demonstrates the utility of the COM-B model for understanding self-isolation behaviour despite the constraints of secondary data. The findings highlight opportunity and motivation as key levers for promoting adherence and offer actionable insights for policymakers to design interventions that enhance motivation, strengthen social support, and sustain compliance during future public health emergencies.

## Introduction

Severe acute respiratory syndrome coronavirus-2 (SARS-CoV-2) is responsible for coronavirus disease (COVID-19). Originating in Wuhan, China, in 2019, the virus spread rapidly worldwide owing to its high transmissibility. The World Health Organization (WHO) declared COVID-19 a pandemic in March 2020^1^. As infections and fatalities sharply increased, public health authorities urgently sought effective strategies to contain transmission, particularly before pharmaceutical interventions became available.

Governments have implemented a range of non-pharmaceutical interventions (NPIs) as the first line of defence. These include lockdowns, quarantine, social distancing, wearing masks, and self-isolation^2,3^. While essential for slowing the spread, their effectiveness relies heavily on public compliance. As Michie noted, “ Behavioural science must be at the heart of the public health response’^4^.

The relationship between behaviour and disease transmission has long been recognised^5,6^. During the 2009 H1N1 pandemic, behavioural variations were shown to influence epidemic trajectories^7–9^. These lessons highlight the need to understand behavioural drivers, particularly when interventions depend on voluntary adherence to them.

In the United Kingdom (UK), self-isolation was a central NPI during the pandemic. Introduced in March 2020 and later mandated by law in September 2020, it requires individuals to remain at home if they are symptomatic or have been in contact with a confirmed case^10,11^. Its success depends on both the willingness and ability to comply with it.

This study applies the COM-B model, a behavioural framework that integrates capability, opportunity, and motivation to explain behaviour^12^. The model identifies psychological and physical capacity (capability), external conditions that enable or constrain action (opportunity), and internal processes that drive behaviour (motivation)^13^. Behaviour results from the dynamic interaction of these components, each of which must be addressed to enable change. Self-isolation is driven by knowledge and mental preparedness (capability), financial or social support (opportunity), and motivations—both reflective (deliberate decisions) and automatic (emotions and habits)—that shape action.

Several behaviour-change models (BCMs) have been applied to understand compliance with COVID-19 measures, notably the Health Belief Model (HBM), Theory of Planned Behaviour (TPB), and Information–Motivation–Behavioural Skills Model (IMB)^14–17^. The HBM emphasises perceived susceptibility, severity, and benefits^18–20^, but its critics argue that it overly focuses on individual cognition, neglecting broader social, structural, and emotional influences^21^. The TPB extends the HBM by incorporating attitudes, subjective norms, and perceived behavioural control^22^, yet research indicates that it often relies on static predictors of intention and may struggle to account for behavioural change when contexts shift rapidly^23,24^. In contrast, the COM-B model situates behaviour within a dynamic system of Capability, Opportunity and Motivation, highlighting how internal and external factors interact and reshape each other under evolving conditions^12^. Its systems-oriented logic makes it particularly suitable for analysing behavioural adaptation during rapidly changing public health crises.

Despite its relevance, the COM-B model has been applied in only a few COVID-19 studies. In the United Kingdom, Gibson Miller et al.^25^ used survey data early in the pandemic to examine hygiene behaviours and found that all three COM-B components predicted adherence, with reflective motivation exerting the strongest influence; psychological capability and social opportunity were also positively associated with hygiene practices. Chater et al*.*^26^ conducted qualitative interviews with employees in high-risk environments such as schools, care homes, and warehouses, applying a combined COM-B and Theoretical Domains Framework (TDF) analysis across multiple preventive behaviours (“Hands, Face, Space, Fresh Air” and “Find, Isolate, Test, Vaccinate”). Their findings underscore the importance of supportive leadership and co-designed interventions that efficiently target shared behavioural barriers across contexts. Similarly, Bonner et al.^27^ mapped behavioural barriers to COVID-19 testing onto the COM-B components in Australia, identifying limitations in capability (e.g. knowledge gaps), opportunity (e.g. access and economic constraints), and motivation (e.g. perceived discomfort and anticipated disruption) as key deterrents to testing. These studies collectively demonstrate the versatility of the COM-B model in capturing behavioural determinants during the pandemic, while highlighting the need for continued model-based analyses using diverse data sources.

This study retrospectively applied the COM-B model to examine the behavioural determinants of self-isolation in the UK between March and May 2020. Using national survey data^28^, this study investigates how capability, opportunity, and motivation interact to influence compliance. Structural equation modelling was used to assess the relationships among these constructs. By identifying behavioural drivers, this study aims to provide insights that can inform communication and engagement strategies, contributing to more effective and sustainable interventions in future public health crises.

## Methods

This study was a retrospective analysis using data from the UK Office for National Statistics (ONS) Opinions and Lifestyle Survey^28^. Although the survey began in 2019, its focus shifted in March 2020 to capture public opinions on COVID-19-related behaviour, including social distancing and self-isolation. The current study focuses on the period between March and May 2020 as shown in Table 1, when specific data were collected on social distancing measures. Participants were randomly selected from individuals who had previously completed other ONS surveys, with each wave comprising approximately 5,000 respondents aged 16 years and above. Waves B to K of the survey were combined, resulting in a pooled dataset of 11,213 responses.

**Table 1:** Survey waves and corresponding data collection periods (March–May 2020) from the ONS Opinions and Lifestyle Survey used in this study.

**Survey**	**Start**	**End**
OPN B	27-Mar	05-Apr
OPN C	03-Apr	12-Apr
OPN D	09-Apr	19-Apr
OPN E	17-Apr	26-Apr
OPN F	24-Apr	03-May
OPN G	30-Apr	10-May
OPN H	07-May	17-May
OPN I	14-May	17-May
OPN J	21-May	24-May
OPN K	28-May	31-May

Each wave represents an independent cross-sectional sample, and no unique identifiers are available to link participants across waves. Consequently, the dataset was treated as cross-sectional, and the structural equation models were estimated at the individual level, without multilevel adjustments. This design captures population-level associations across waves rather than changes within a person over time.

For this study, only a subset of responses was suitable for analysis. Because the survey was not originally designed to measure behavioural constructs from the COM-B model, a substantial number of responses were excluded during the data preparation. Specifically, all responses marked as “Not applicable”, “Don’t know”, “Prefer not to say”, “Refusal”, or those with missing values were removed. These options did not contribute meaningful information to the rationalisation of COM-B constructs and would have introduced noise or ambiguity into model estimation.

A rigorous approach to missing data was adopted: any observation with incomplete responses to variables necessary for COM-B parameterisation was excluded. This strict handling of missing data ensured that the final analytical sample comprised only complete cases suitable for reliable model estimation. After these exclusions, the final sample consisted of 1,656 observations. Detailed distribution of participants are presented in Table 2. While this represented a notable reduction from the initial dataset, it reflected a necessary trade-off in using secondary data that were not originally intended for this theoretical framework. Although participants with incomplete responses were excluded from the analytical sample, this may have introduced some degree of selection bias. However, given that the Opinions and Lifestyle Survey uses a stratified random sampling design and national weighting to ensure representativeness, any resulting bias is expected to be limited in this study.

While sample exclusion may introduce nonresponse bias, evidence shows that response rates alone are not reliable indicators of bias magnitude and that associations between behavioural variables may remain robust even when prevalence estimates are affected^29–31^.

Therefore, the resulting sample was more robust and appropriate for the study’s analytical goals. Furthermore, we used composite reliability (CR) to assess the internal consistency of the latent constructs. Unlike Cronbach’s alpha, which assumes equal indicator loadings, CR incorporates the actual factor loadings and measurement error from the model, providing a more accurate estimate of how well the observed items represent their underlying construct.

The survey items used in this analysis (Table 3) were identified from the publicly available questionnaire of the Opinions and Lifestyle Survey (2019) conducted by the UK Office for National Statistics^28^. To assign each item to the appropriate COM-B construct—Capability, Opportunity, or Motivation—a structured elicitation process was conducted. Two independent experts in behavioural science (the co-author) and the lead author individually reviewed and assigned each survey item to the COM-B component they deemed most appropriate. The outputs from each reviewer were collated, and discrepancies in item classification were noted.

To resolve these differences, follow-up elicitation meetings were held, during which the rationale behind each reviewer’s classification was discussed. Through consensus, final assignments were made for each item, ensuring alignment with the theoretical definitions of the COM-B Model. The final categorisations, along with the justifications for each assignment, are presented in Table 3. This mapping was essential for aligning the selected items with the theoretical components of the COM-B model and ensuring the conceptual validity of the constructs used in the study.

**Table 2:** Distribution of Participants by Region, Educational Level, and Age Group (N = 1656).

**Group**	**Region (n)**	**Education level (n)**	**Age group (n)**
1	79	826	66
2	183	208	71
3	155	163	115
4	111	91	150
5	130	168	196
6	144	87	228
7	121	45	174
8	252	68	209
9	164	–	180
10	143	–	142
11	174	–	66
12	–	–	35
13	–	–	24
**Total**	**1656**	**1656**	**1656**

**Table Key:** Group numbers correspond to specific categories in each variable as follows.

**Regions:** 1 = North East, 2 = North West, 3 = Yorkshire & The Humber, 4 = East Midlands, 5 = West Midlands, 6 = East of England, 7 = London, 8 = South East, 9 = South West, 10 = Wales, 11 = Scotland.

**Educational Levels:** 1 = Degree-level qualification, 2 = Higher education below degree, 3 = A-levels or equivalent, 4 = ONC/National Level BTEC, 5 = O’Level or GCSE equivalent, 6 = GCSE grades D–G or equivalent, 7 = Other qualifications, 8 = No formal education.

**Age Groups:** 1 = 16–19, 2 = 20–24, 3 = 25–29, 4 = 30–34, 5 = 35–39, 6 = 40–44, 7 = 45–49, 8 = 50–54, 9 = 55–59, 10 = 60–64, 11 = 65–69, 12 = 70–74, 13 = 75+.

“–” indicates missing or inapplicable data.

This study focuses on the period corresponding to the initial implementation and gradual easing of lockdown restrictions in the United Kingdom. The first national lockdown was announced by the UK Prime Minister on 23 March 2020 and came into legal effect on 26 March. The restrictions were extended on 16 April for an additional three weeks, and by 10 May, a conditional plan for easing the lockdown was introduced, allowing those unable to work from home to resume work^32^. Therefore, the analysis in this study focuses on this critical period—from the start of the lockdown to the early phase of reopening—as outlined in Table 1.

### COM-B operationalisation

The survey items were mapped onto the COM-B constructs through a multi-step process. First, we reviewed the theoretical definitions of capability, opportunity, and motivation as described by Michie et al. (2011)^12^, focusing on their behavioural relevance in the context of preventing and controlling infectious diseases. Each ONS item was assessed for conceptual alignment with the COM-B components. For example, items capturing knowledge or informational sufficiency were mapped to capability (psychological capability), items reflecting exposure to others’ behaviour were mapped to opportunity (social opportunity), and items related to health status or worry were mapped to motivation (reflective motivation associated with perceived vulnerability). These assignments were independently reviewed by three behavioural science researchers and refined through consensus discussions. To enhance transparency, we provide expanded theoretical and empirical justifications for each mapping decision in the supplementary material.

In addition to the core COM-B pathways, an exploratory path from Opportunity to Capability was included. This decision was informed by emerging empirical evidence showing that external opportunities can shape individuals’ perceived capability by enhancing access to supportive environments, resources, or behavioural models. Willmott et al. (2021) ^33^, for instance, reported a similar opportunity, capability, motivation, behaviour sequence in their application of COM-B, illustrating how social and contextual opportunities can strengthen perceived ability to perform a behaviour. Incorporating this path therefore aligns with contemporary extensions of COM-B in applied public health research.

**Table 3:** Survey items mapped to COM-B constructs for self-isolation behaviour during COVID-19. Source: Office of National Statistics, Opinions and Lifestyle Survey (2024)^28^.

**COM-B component**	**Survey item**	**Response options (coded)**	**Rationale for variable**
Capability	How often do you feel lonely? (COV_Lon)	Often/always (1), Some of the time (2), Occasionally (3), Hardly ever (4), Never (5)	Frequent loneliness may reduce mental capacity for isolation
Capability	Do you feel like you have enough information about how to protect yourself? (COV_ProInfo)	Yes (2), No (1)	Awareness improves capability for self-protection
Capability	Do you feel like you have enough information about the UK’s pandemic response? (COV_UKInfo)	Yes (2), No (1)	Awareness of government measures increases behavioural readiness
Opportunity	Has anyone in your household self-isolated recently? (COV_HHIso)	Yes (2), No (1)	Seeing others self-isolate promotes shared norms
Opportunity	Do you know someone outside your household who has self-isolated? (COV_OutIso)	Yes (2), No (1)	Exposure to others’ behaviours reinforces opportunities
Motivation	How is your health in general? (Qhealth)	Very good (5) to Very bad (1)	Perceived vulnerability influences isolation decisions
Motivation	Do you have any physical or mental health conditions? (Healill)	Yes (2), No (1)	Health risk perception influences self-protection behaviour
Motivation	How worried are you about COVID-19’s impact? (COV_Wor)	Very worried (5) to Not worried at all (1)	Greater concern may motivate isolation behaviour
Behaviour	Have you self-isolated in the past 7 days? (COV_SelfIso)	Yes (1), No (0)	Main outcome variable

### Data source and design

The Opinions and Lifestyle Survey (OPN), a long-running, extensive survey conducted by the UK Office for National Statistics (ONS) to gather public attitudes and behaviours on matters of national interest, was used in this study. The OPN used a stratified random sampling technique to choose one adult ($\ge$ 16 years of age) from every private home in Great Britain. Data were collected through a mixed-mode process that combined online self-completion with telephone interviews, where needed. In March 2020, the OPN was adapted into a weekly survey to monitor the social and behavioural impacts of the COVID-19 pandemic. Each wave represents an independent cross-sectional sample of approximately 1,000 to 4,500 adults drawn from households that had previously participated in ONS social surveys. The COVID-19 module includes standard questions on health, risk perception, and lifestyle behaviours, together with new items specific to the pandemic. The dataset analysed in this study is part of the Secure Access study (SN 8635), and full methodological documentation is available through the ONS Quality and Methodology Information reports. ONS population weights were applied to ensure representativeness of the sample. Because respondents cannot be linked across waves, data collected between March and May 2020 were pooled to provide a cross-sectional snapshot of the early pandemic responses.

### Statistical analysis

Structural equation modelling (SEM) was used to retrospectively examine how capability, opportunity, and motivation influenced self-reported behavioural adherence to social distancing during the COVID-19 pandemic. Data were obtained from the Office of National Statistics. (2024). ONS Opinion and Lifestyle Survey, 2019-2023: secure Access. [data collection]. (11th Edition) SN: 8635, DOI: http://doi.org/10.5255/UKDA-SN-8635-11, collected by the UK Office for National Statistics (2024)^28^. Responses marked as “Not applicable”, “Don’t know”, “Prefer not to say”, “Refusal”, or with no response were considered non-contributory to the analysis of COM-B constructs and were excluded. Following this exclusion, the final analytic sample comprised 1,656 complete cases. Survey items mapped to the COM-B components were scored on a scale of 1–5, with higher scores reflecting greater capability, opportunity, or motivation. Behavioural adherence was measured as a binary outcome, with 1 indicating self-reported adherence and 0 indicating non-adherence to social distancing guidelines. Statistical analyses, including the computation of means and standard deviations, were conducted using R.

**Table 4:** Descriptive statistics for COM-B components and self-reported behavioural adherence. Values represent the mean and standard deviation for each construct (N = 1656).

**COM-B component**	**Mean (SD)**
Capability	7.21 (1.31)
Opportunity	2.69 (0.69)
Motivation	6.85 (1.48)
Behaviour	0.17 (0.37)

## Results

### Preliminary analysis and model evaluation

Table 4 displays the mean and standard deviation for each COM-B construct and behavioural outcome variable. Capability had the highest mean score (M = 7.21, SD = 1.31), followed by motivation (M = 6.85, SD = 1.48) and opportunity (M = 2.69, SD = 0.69), suggesting a generally high perceived ability and willingness to self-isolate during the pandemic. The behavioural adherence variable had a mean (M = 0.17, SD = 0.37), indicating that only a small proportion of respondents reported engaging in self-isolation during the study period.

A structural equation model (SEM) was applied to assess the interrelationships among the COM-B constructs, with latent relationships specified between the survey items and their corresponding COM-B components (Table 3). SEM was selected because it enables the simultaneous estimation of multiple associations, combining factor analysis and multiple regression to evaluate both measurement and structural components within one framework. This allowed assessment of the relationships among capability, opportunity, motivation, and self-isolation behaviour, including the possibility of indirect pathways suggested by the structural model.

The maximum likelihood method was used for the model estimation. Model fit was evaluated using the Comparative Fit Index (CFI) and Tucker–Lewis Index (TLI), with values $\ge 0.95$ indicating good fit and $\ge 0.90$ indicating acceptable fit, and the Root Mean Square Error of Approximation (RMSEA) and Standardised Root Mean Square Residual (SRMR), with values $\le 0.05$ indicating good fit and $\le 0.08$ indicating acceptable fit^34–36^.

**Fig. 1:**
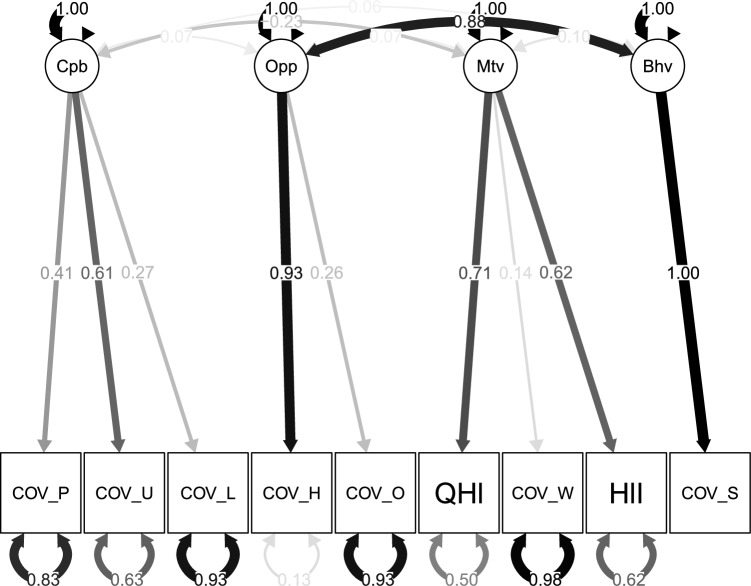
Confirmatory factor analysis (CFA) model showing the latent constructs (Cpb, Opp, Mtv, Bhv) and their observed indicators, with standardized factor loadings and correlations among latent variables.**Note:** COV_Lon = loneliness; COV_ProInfo = information on self-protection; COV_UKInfo = information on UK response; COV_HHIso = household self-isolation; COV_OutIso = outside household self-isolation; Qhealth = self-rated health; Healill = existing health condition; COV_Wor = worry about COVID-19; COV_SelfIso = self-isolation behaviour.

Figure 1 presents the confirmatory factor analysis (CFA), which illustrates how well the observed variables represent their underlying theoretical constructs within the COM-B framework. For the Capability construct, the three observed indicators had standardised loadings of 0.41, 0.61, and 0.27, indicating that the latent factor explained a moderate proportion of variance in the first two indicators but a weaker proportion in the third indicator. Similarly, the Opportunity construct included two indicators with loadings of 0.93 and 0.26, suggesting that while one item strongly represented the construct, the other contributed less to it. The Motivation construct comprised three indicators with loadings of 0.71, 0.14, and 0.62, showing moderate to strong associations for two items and a weaker contribution from the third. Average variance extracted (AVE) values were calculated and are reported in the Supplementary Materials to provide additional information on convergent validity, recognising that the indicators were not originally designed as psychometric scales.

While some indicators demonstrated low loadings, these results are not unexpected, given the use of secondary data that were not originally designed to measure the COM-B constructs directly. The observed variation likely reflects limitations in item specificity rather than a theoretical inconsistency. Taken together, the CFA provides partial but meaningful support for the proposed measurement model, indicating that the selected indicators capture the key dimensions of capability, opportunity, and motivation within the constraints of the available data. These results reinforce the exploratory nature of the present analysis, positioning it as a valuable foundation for future studies employing purpose-built instruments to test the COM-B model more comprehensively.

### Trends in COM-B constructs

**Fig. 2:**
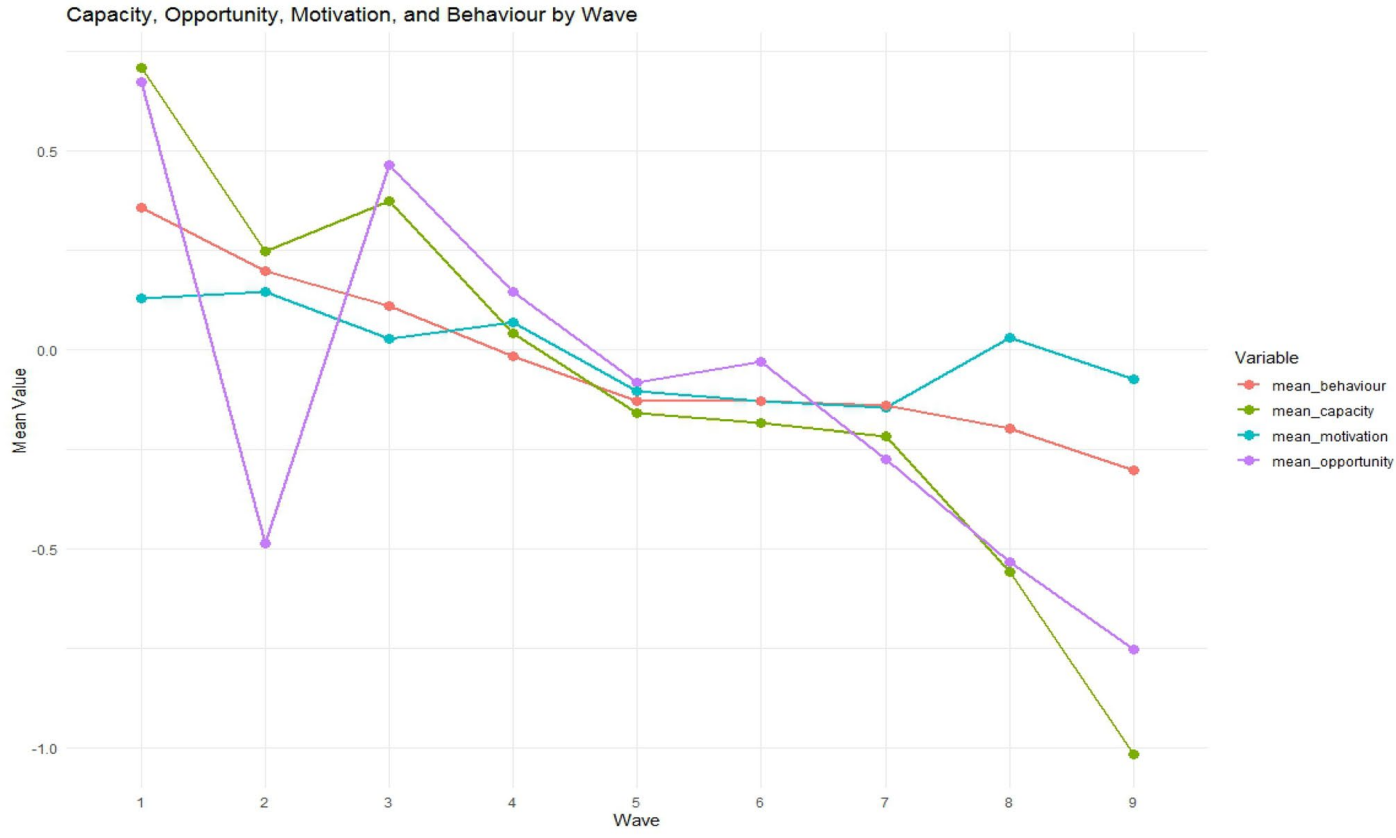
Descriptive trends in the COM-B constructs and self-isolation behaviour across survey waves (March–May 2020).

Figure 2 shows the trends in capability, opportunity, motivation, and self-isolation behaviour between 27th March and 31 May 2020. These temporal patterns are presented as descriptive summaries based on standardised mean scores and are not intended to imply a statistically significant change over time. The visual trends provide contextual insight into how reported capability, opportunity, motivation, and behaviour fluctuated during the different phases of the early pandemic response.

All COM-B components declined over time, particularly after wave 4. Capability and opportunity showed the steepest reductions from Wave 6 onwards, reaching their lowest points in Wave 9. Motivation remained comparatively stable, with minor fluctuations, whereas behavioural adherence decreased steadily over time. These trends coincided with the initial UK lockdown (23rd March–10th May) and its phased easing thereafter^32^, suggesting a gradual reduction in compliance as restrictions persisted.

### Structural equation modelling

**Fig. 3:**
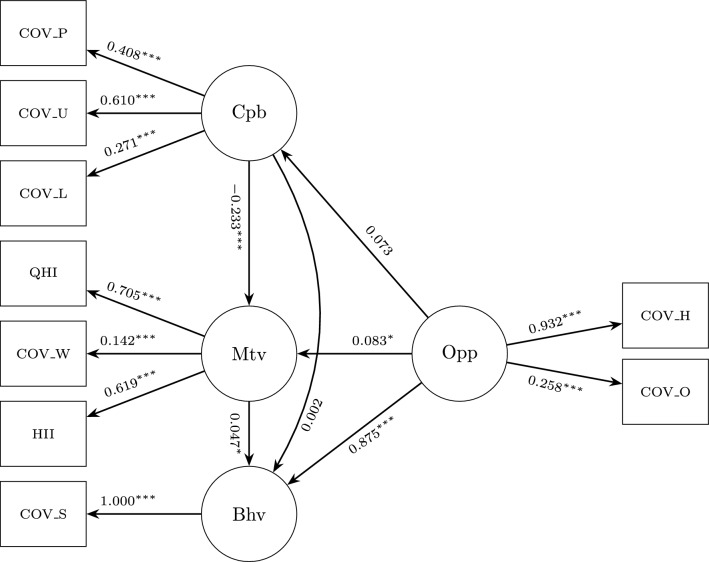
Structural equation model of relationships between COM-B components and self-reported self-isolation behaviour during the COVID-19 pandemic. The standardised regression coefficients and significance levels are shown for each path. $$(N = 1656)$$; $$^*p < 0.05$$, $$^{**}p < 0.01$$, $$^{***}p < 0.001$$. **Note:** COV_Lon = loneliness; COV_ProInfo = information on self-protection; COV_UKInfo = information on UK response; COV_HHIso = household self-isolation; COV_OutIso = outside-household self-isolation; Qhealth = self-rated health; Healill = existing health condition; COV_Wor = worry about COVID-19; COV_SelfIso = self-isolation behaviour.

The structural equation model (SEM) examined the pattern of associations among COM-B constructs, including pathways that suggest potential indirect relationships (Fig. 3). All latent constructs were measured using multiple indicators, and the standardised regression coefficients are presented in Table 5 and Fig. 3. As previously stated, the COM-B components in this study were derived from secondary data items that were not originally designed to measure the model. Although this means that the results should be interpreted as indicative rather than precise psychometric estimates, the approach is nevertheless informative. Mapping existing items onto COM-B constructs highlights useful relationships between capability, opportunity, motivation, and behaviour that can inform future intervention planning, as well as the monitoring and evaluation of behavioural interventions.

**Table 5:** Standardized regression estimates for Motivation, Capability, Opportunity, and Behavior.

		std.all ($\beta$)	S.E.	p
Motivation	Capability	−0.233	0.050	.000
Motivation	Opportunity	0.083	0.035	.015
Capability	Opportunity	0.073	0.039	.061
Behav	Motivation	0.047	0.040	.017
Behav	Opportunity	0.875	0.255	.000
Behav	Capability	0.002	0.048	.933
Capability	COV_ProInfo	0.408	0.012	.000
Capability	COV_UKInfo	0.610	0.030	.000
Capability	COV_Lon	0.271	0.041	.000
Opportunity	COV_HHIso	0.932	0.013	.000
Opportunity	COV_OutIso	0.258	0.013	.000
Motivation	QHealth	0.705	0.044	.000
Motivation	COV_Wor	0.142	0.030	.000
Motivation	HealIll	0.619	0.025	.000
Behav	COV_SelfIso	1.000	0.019	.000

*Note.* Standardised regression coefficients ($\beta$) are reported. S.E. = Standard Error. p-values: values reported as *.000* indicate $p < .001$.

The composite reliability values for the latent constructs were 0.27 for Capability, 0.47 for Opportunity, and 0.39 for Motivation. While these estimates fall below the conventional thresholds for internal consistency, this is likely due to the use of secondary data, where the available indicators may not have been designed to fully capture the theoretical dimensions of each construct. However^37^,,notes that conventional reliability thresholds are historically contingent benchmarks rather than psychometric absolutes, and should be interpreted within the context of measurement model specification and study design. In this case, low reliability reflects retrofitting existing survey items onto COM-B constructs rather than purpose-built measurement. Despite these measurement limitations, the structural model demonstrated strong explanatory power ($R^2 = 0.773$) and good overall fit (RMSEA = 0.049, CI: 0.040–0.058; CFI = 0.966; TLI = 0.944; SRMR = 0.040), indicating the predictors effectively captured key behavioural determinants. This suggests that predictive utility and theoretical alignment serve as meaningful indicators of model adequacy when purpose-built measures are unavailable. Accordingly, the results should be viewed as indicative rather than confirmatory, underscoring the importance of interpreting reliability measures within their methodological context.

#### Key pathways

In the structural equation model, capability was represented by information-related and psychosocial items, including access to protective information, knowledge of the UK’s pandemic response and experiences of loneliness. All indicators loaded significantly and positively on the construct, with knowledge of the UK’s pandemic response ($\beta = 0.610$, $p <.001$) and access to self-protection information ($\beta = 0.408$, $p <.001$) being the strongest contributors. Loneliness also loaded significantly ($\beta = 0.271$, $p <.001$), underscoring the importance of psychological well-being and communication in shaping perceived capability. At the structural level, however, capability did not have a direct association with behaviour ($\beta = 0.002$, $p =.933$) in this study. Instead, it was indirectly linked to self-isolation through its negative association with motivation ($\beta = -0.233$, $p <.001$), though formal testing would be needed to confirm causality.

Opportunities were captured by witnessing self-isolation within or outside the household. Household self-isolation was the strongest indicator ($\beta = 0.932$, $p <.001$), whereas awareness of self-isolation outside the household contributed positively but less strongly ($\beta = 0.258$, $p <.001$). At the structural level, opportunity demonstrated the most powerful direct effect on behaviour ($\beta = 0.875$, $p <.001$), highlighting that social and environmental contexts, particularly within the home, play a central role in enabling adherence to isolation.

Motivation was defined by health-related and emotional indicators, all of which were significant. Self-rated health was the strongest contributor ($\beta = 0.705$, $p <.001$), followed by the presence of a physical or mental health condition ($\beta = 0.619$, $p <.001$) and worry about the impact of COVID-19 ($\beta = 0.142$, $p <.001$). Motivation also had a significant, though modest, direct effect on behaviour ($\beta = 0.047$, $p <.05$), and the model indicated that capability was associated with behaviour indirectly through its association with motivation; however, this reflects a suggested pathway rather than a formally tested mediation effect.

Overall, the structural model explained $77.3\%$ of the variance in self-isolation behaviour ($R^{2} = 0.773$), highlighting the strong explanatory power of the COM-B components when applied to population-level survey data. Although the overall measurement model showed an acceptable fit, several indicators showed weaker factor loadings, reflecting variation in how strongly individual items captured their intended COM-B constructs. These uneven loadings are expected given that the survey items were not originally designed to operationalise COM-B domains, and therefore the results should be viewed as indicative of broad behavioural patterns rather than precise measurement of each construct.

## Discussion

This study examined the behavioural factors that influenced self-isolation during the COVID-19 pandemic in the United Kingdom. The findings show that opportunity was the strongest predictor of self-isolation, whereas motivation played a smaller but significant role. Capability, on the other hand, had no direct effect but influenced behaviour indirectly through its negative relationship with motivation. These results contribute to the knowledge through the application of structural equation modelling (SEM) with the COM-B framework at the national level. We highlight how environmental and psychological factors interact to shape compliance during health crises.

Rather than establishing a new methodological precedent, the contribution lies in showing how behavioural theory can be operationalised in existing surveillance datasets to generate timely evidence during health crises. By identifying the strongest influences, the model helps policymakers design more targeted interventions. Importantly, this approach not only explains why certain health behaviours are adopted, but also uncovers the causal pathways through which they can be influenced or sustained. Health communication and intervention strategies can be more precisely aligned with the psychological and contextual determinants of compliance.

Regarding capability, knowledge of the UK’s pandemic response ($\beta = 0.610$, $p <.001$) and access to self-protection information ($\beta = 0.408$, $p <.001$) were the primary contributors to this construct, while loneliness showed weaker contribution ($\beta = 0.271$, $p <.001$). Despite these information-related indicators loading strongly, capability did not directly translate into higher adherence. Instead, capability was negatively associated with motivation, which, in turn, reduced the likelihood of self-isolation. This contrasts with earlier studies that identified capability as a direct enabler of protective behaviour^10,11,38^. One explanation may be that when information becomes overly abundant, individuals reach a saturation point in their ability to process and apply it. As shown by^39^, excessive media and social information on COVID-19 can overwhelm individuals, causing their capacity to absorb new information to become stagnant. In such situations, information overload may be associated with reduced motivation, rather than enhanced motivation. This suggests that simply increasing the flow of information is insufficient and may even be counterproductive unless combined with motivational and structural support that helps individuals translate information into action.

In contrast, it is also important to consider the quality and consistency of the information individuals receive, not only its volume. During the early stages of the pandemic, information sources were diverse and abundant, and some were contradictory, with changing guidance on risk levels, isolation rules, and transmission modes^40^. Such inconsistencies may have created confusion and diminished people’s confidence in official communication. Studies have shown that when public messages are inconsistent, ambiguous, or change rapidly, trust in authorities tends to decline, and the motivation to act may weaken—even among individuals who perceive themselves as capable^40–42^. This suggests that the negative linkage between capability and motivation observed in this study may reflect not only information overload but also message inconsistency and diminished trust, which together undermine motivational engagement.

Opportunity was the strongest factor associated with self-isolation ($\beta = 0.875$, $p <.001$). However, the two opportunity indicators differed markedly: household self-isolation was the dominant contributor ($\beta = 0.932$, $p <.001$), while awareness of self-isolation outside the household contributed far less ($\beta = 0.258$, $p <.001$). This substantial difference indicates that immediate household modeling had considerably greater influence than observing others in broader social networks. This pattern aligns with research by Putu A. I. *et al.*^43^, who found that adherence was strongly influenced by compliance within one’s immediate environment. However, our findings suggest this effect operates primarily at the household level rather than through wider peer networks. The evidence here suggests that persuasive strategies would be most effective when they emphasize household cohesion rather than broader community peer influence. The opportunity construct in this study reflects social rather than physical opportunities, and specifically household-level rather than community-level social influence. While two indicators captured observed self-isolation behaviour, household exposure ($\beta = 0.932$) dominated over outside-household awareness ($\beta = 0.258$). The findings should therefore be interpreted as indicative of normative household opportunity rather than broader peer or community influence. This distinction is important: the results suggest that physical proximity and shared living spaces drive adherence more than observation of behaviours in wider social networks.

Motivation also mattered, though to a lesser degree than opportunity. However, indicator contributions varied considerably: self-rated health ($\beta = 0.705$, $p <.001$) and existing health conditions ($\beta = 0.619$, $p <.001$) were strongly associated with motivation to self-isolate, while worry about COVID-19 showed a much weaker contribution ($\beta = 0.142$, $p <.001$). This pattern indicates that tangible health vulnerability—rather than emotional concern—was the primary motivational driver. These findings align with evidence showing that perceived personal health risk boosts motivation for preventive actions such as self-isolation^44^. The motivation indicators used in this study—general health status, existing health conditions, and worry about COVID-19—primarily captured self-oriented motivations related to personal health and perceived vulnerability. However, given the weak loading of worry, interpretations should focus primarily on health-status-driven motivation rather than emotional or affective responses. Therefore, these measures reflect individual protective concerns rather than prosocial motivation or collective responsibility. While motivation was directly associated with adherence, this interpretation should be understood within the scope of self-focused motivations, and it is not possible to infer if prosocial motivations, such as concern for protecting others, would operate the same way based on the present data. Motivation was also associated with both capability and behaviour, suggesting a possible indirect pathway consistent with the COM-B proposition that capability may influence action through motivational processes. Although this indirect pathway was not formally tested, the observed associations suggest a plausible theoretical link. Interventions that address both emotional drivers and structural support may help strengthen compliance indirectly by boosting motivation, though the evidence here suggests such interventions should prioritize those with poor health status rather than relying primarily on heightening emotional worry.

The behavioural indicator used in this study—self-isolation within the past seven days—captures a specific and time-bound adherence form. However, this measure should be interpreted within the policy context of the study period (March–May 2020), when public health guidance in the United Kingdom evolved rapidly from nationwide lockdown to phased easing of restrictions. During this transition, the requirement to self-isolate primarily applied to individuals with symptoms, those living with symptomatic persons, and those identified as close contacts. Consequently, non-isolation among some respondents may not necessarily indicate non-compliance but rather the non-applicability of the guidance at that time. This contextual nuance reinforces the interpretation of behavioural outcomes as indicative of adherence tendencies rather than a universal measure of compliance. Future studies should incorporate multiple behavioural indicators (e.g. distancing, mask-wearing, or hand hygiene) to provide a more comprehensive picture of preventive behaviour over time.

It is also important to note that the dataset used does not record the reasons why individuals chose to self-isolate. Previous research indicates that isolation decisions in the early stages of the pandemic were influenced by a range of factors, including the perceived risk of infection, a sense of duty to protect others, and practical or social limitations, rather than solely by confirmed exposure^11,45^. Because the survey did not measure symptoms, test results, or contact with confirmed cases, we could not determine whether reported self-isolation reflected exposure-driven requirements, precautionary behaviour, or normative social influences. Therefore, the findings should be interpreted as reflecting patterns of reported isolation rather than the underlying motivations for that behaviour.

This study had several limitations. First, the study combined multiple survey waves into a single cross-sectional dataset, which limited ability to examine behavioural changes over time. Because analysis was cross-sectional, observed associations cannot be interpreted as evidence of causality. While SEM allows directional path specification consistent with theory, it does not establish temporal precedence or rule out reverse causation. These relationships should be understood as patterns of association rather than causal effects. Longitudinal or experimental designs are necessary to establish whether COM-B component changes precede and produce behavioural change. Therefore, the relationships between capability, opportunity, motivation, and behaviour should be understood as indicative patterns rather than directional effects. Future research should employ longitudinal analyses to assess how the COM-B components evolve across different phases of a pandemic, thereby enabling real-time behavioural monitoring and adaptive interventions. Second, the survey was not originally designed to be directly mapped onto the COM-B framework. Consequently, some constructs may be under-represented, limiting the model’s theoretical granularity.

Additionally, variation in indicator loadings means not all items contributed equally to their constructs. Within motivation, emotional worry ($\beta = 0.142$) contributed far less than health status indicators ($\beta = 0.705$, 0.619); within opportunity, outside-household exposure ($\beta = 0.258$) was substantially weaker than household exposure ($\beta = 0.932$); and within capability, loneliness ($\beta = 0.271$) contributed less than information access ($\beta = 0.408$, 0.610). Consequently, interpretations emphasizing household influence over broader peer networks, health vulnerability over emotional concern, and information access over psychological isolation more accurately reflect the data. An item-level logistic regression approach could complement these findings by identifying which individual survey items most directly predict self-isolation, independent of their theoretical grouping, thereby distinguishing the explanatory value of the COM-B framework from the predictive power of specific indicators.

The dataset lacks direct measures of personal COVID-19 infection or exposure, which limits the ability to distinguish social opportunity from potential exposure-driven self-isolation. Although the opportunity indicators capture reported awareness of others’ self-isolation, this may partially overlap with exposure pathways during the early pandemic period. The findings should therefore be interpreted as indicative of perceived social opportunity rather than verified exposure-related behaviour. Future studies would benefit from purpose-built instruments that explicitly measure COM-B domains, including subcomponents such as types of social support (emotional, financial, and instrumental) and forms of motivation (intrinsic versus extrinsic).

It is also important to acknowledge that the COM-B constructs are multidimensional by design. The present operationalisation captures only a subset of these dimensions: social rather than physical opportunity and reflective rather than automatic motivation^12^. These omissions stem from the constraints of secondary data, rather than theoretical oversight. Future primary studies should therefore include purpose-built items that measure the full spectrum of COM-B processes, such as environmental or infrastructural opportunity and automatic motivational pathways, such as habit and emotional salience.

While item-level approaches such as logistic regression can identify the strongest individual predictors of behaviour, they are less well suited for studies grounded in a theoretical framework such as COM-B, as they treat each item as an independent determinant rather than an indicator of an underlying construct. Given that the ONS items were not designed to function as standalone behavioural predictors and varied in conceptual scope, analysing them individually could risk over-interpreting single indicators—particularly where constructs were represented by only one or two items. Therefore, we retained a latent-construct approach aligned with COM-B, while acknowledging that item-level predictive analyses may be more appropriate in future primary data collections developed specifically to measure capability, opportunity, and motivation.

In summary, this study found that opportunity and motivation were significantly associated with self-isolation in cross-sectional analysis, whereas capability showed an indirect association through motivation. By applying the COM-B model to national survey data, the findings suggest clear areas for public health action: strengthening household-level behavioural modeling (rather than broader peer networks), targeting health-vulnerable populations for motivational support, and ensuring information provision is accompanied by structural and emotional support. However, these policy-oriented recommendations should be viewed as theoretically informed reflections derived from observed associations rather than empirically tested interventions. While the analysis identified household-level opportunity and health-based motivation as key correlates of adherence, the study did not directly measure social support mechanisms, financial incentives, or policy structures. The suggestions to strengthen household-based interventions and combine motivational with structural supports are therefore intended as conceptual extensions consistent with the COM-B framework, highlighting potential directions for policy and intervention design rather than confirmed causal levers. These patterns align with COM-B theoretical propositions, though causal inference requires longitudinal or experimental confirmation. These insights can help shape communication and policy responses to future public health emergencies.

More broadly, this secondary data approach illustrates both the utility and limits of mapping existing survey instruments onto behavioural frameworks such as COM-B. Therefore, future research should prioritise developing purpose-built COM-B measures that explicitly capture its multidimensional structure and can be validated prospectively in pandemic and non-pandemic contexts.

## Supplementary Information

Supplementary Information.

## Data Availability

The data that support the findings of this study are available from UK Data service: Office of the National Statistics. (2024). ONS Opinion and Lifestyle Survey, 2019-2023: secure Access. [data collection]. 11th Edition. UK Data Service. SN: 8635, DOI: http://doi.org/10.5255/UKDA-SN-8635-11. Restrictions apply to the availability of these data, which were used under license for the current study, and so are not publicly available. However, the data and code are available with permission from the UK Data Service.
