# Supplementary material for: Assessing the relationships between capability, opportunity, and motivation in influencing self-isolation behaviour during pandemics
Source: Sci Rep. 2026 Jan 14;16:5251. doi: 10.1038/s41598-026-36198-7 (PMC12881627; doi:10.1038/s41598-026-36198-7)
Supplement: Supplementary file 1 — Supplementary Information. [file 41598_2026_36198_MOESM1_ESM.pdf]

# Assessing the Relationships Between Capability, Opportunity, and Motivation in Influencing Self-Isolation Behaviour During Pandemics: Supplementary Information

Gbeminiyi J Oyedele[1,2*], Ankit Shanker[1,2], Michael J Tildesley[1,4], Ivo Vlaev[3]

[1*]Institute for Global and Pandemic Planning, University of Warwick,CV4 7AL, Coventry , United Kingdom.
[2]Warwick Medical School, University of Warwick, CV4 7AL, Coventry, United Kingdom.
[3]Centre for Behavioural and Implementation Science Intervention (BISI). Yong Loo Lin School of Medicine, National University of Singapore, 117597, , Singapore.
[4]School of Life science and Mathematics, University of Warwick, CV4 7AL, Coventry, United Kingdom.

*Corresponding author(s). E-mail(s): gbeminiyi.oyedele@warwick.ac.uk;

## Appendix A COM-B Operationalisation Table

| COM-B Component | Survey Item | Response Options (coded) | Extended Theoretical and Empirical Justification |
|---|---|---|---|
| **Capability** | How often do you feel lonely? (COV_Lon) | Often/always (1), Some of the time (2), Occasionally (3), Hardly ever (4), Never (5) | Loneliness influences cognitive capacity, psychological resilience, and the ability to engage in sustained protective behaviours. COM-B defines psychological capability to include emotional and cognitive functioning, which can be undermined by loneliness. Loneliness has been shown to worsen mental health and reduce emotional resilience during the pandemic, which may indirectly affect the cognitive and psychological resources needed for sustained adherence Killgore et al., 2020 [1]. Therefore, this item reflects an emotional–cognitive barrier to behavioural capability. |
| **Capability** | Do you feel like you have enough information about how to protect yourself? (COV_ProInfo) | Yes (2), No (1) | Psychological capability explicitly includes knowledge about what actions are required and how to undertake them. Michie et al. (2011) [2] identify "knowledge" as a core indicator of capability within COM-B. |

*Continued on next page*

| COM-B Component | Survey Item | Response Options (coded) | Extended Theoretical and Empirical Justification |
|---|---|---|---|
| **Capability** | Do you feel like you have enough information about the UK's pandemic response? (COV_UKInfo) | Yes (2), No (1) | Understanding policy measures enhances individuals' ability to translate guidance into action. Within the COM-B framework, capability includes not only cognitive skills but also the comprehension of rules and the contextual conditions under which behaviours should occur. Evidence from recent COVID-19 research shows that when public-health communication is clear, consistent and trustworthy, people report a greater sense of ability to follow restrictions Williams et al., 2023 [3]. Conversely, qualitative work during the pandemic indicates that unclear or shifting guidance made it harder for individuals to understand what was required of them, reducing their perceived ability to adhere Benham et al., 2021 [4]. This item therefore reflects policy-related informational capability—how well individuals feel equipped to act in line with evolving public-health measures. |

*Continued on next page*

| COM-B Component | Survey Item | Response Options (coded) | Extended Theoretical and Empirical Justification |
| --- | --- | --- | --- |
| **Opportunity** | Has anyone in your household self-isolated recently? (COV_HHIso) | Yes (2), No (1) | COM-B conceptualises opportunity as the social or physical environment that enables behaviour. Observing others' behaviour within close social networks is a strong form of social opportunity, shaping perceived norms, modelling, and behavioural feasibility. Social contagion literature shows that witnessing household members perform a behaviour increases one's likelihood of doing the same Christakis & Fowler, (2013) [5]. Seeing household members or people outside the home self-isolate can make individuals more likely to do the same. This shows the power of social connection and peer influence in supporting adherence. Research by Putu A.I. et al. [6] highlights the connection between individual adherence and the compliance of those in their surrounding environment. They highlighted that adherence increases when others around individuals comply. Empirical evidence further indicates that adherence often reflects perceived behaviour within close social circles: people follow guidelines most when they believe their peers do so as well [7]. This item reflects proximal social opportunity. |

*Continued on next page*

| COM-B Component | Survey Item | Response Options (coded) | Extended Theoretical and Empirical Justification |
|---|---|---|---|
| **Opportunity** | Do you know someone outside your household who has self-isolated? (COV_OutIso) | Yes (2), No (1) | Exposure to behaviour in wider social networks reinforces social norms, subjective expectations, and perceived acceptability—all key aspects of social opportunity in COM-B. Prior COVID-19 studies show that knowing others who adhere increases perceived social approval and likelihood of compliance Indrayathi et al., (2021) [6]. This item captures distal social opportunity. |
| **Motivation** | How is your health in general? (Qhealth) | Very good (5) to Very bad (1) | Reflective motivation includes beliefs about one's vulnerability and the perceived value of engaging in a behaviour. Poorer perceived health increases perceived susceptibility, which is a major driver of motivation to adopt self-protective behaviours. Studies confirm that individuals who view themselves as vulnerable show stronger motivation to isolate (Smith et al., 2022; Dryhurst et al., 2020). This item captures self-focused motivational drivers. |
| **Motivation** | Do you have any physical or mental health conditions? (Healill) | Yes (2), No (1) | Underlying health conditions shape perceived personal risk and therefore reflective motivation. COM-B defines reflective motivation to include evaluations of vulnerability and anticipated outcomes of action. Studies by Smith et al., 2020 [8] and Wright et al., 2021 [9] show that individuals with underlying conditions report higher perceived risk and greater adherence to isolation and shielding recommendations. This item therefore reflects risk-based reflective motivation. |

*Continued on next page*

| COM-B Component | Survey Item | Response Options (coded) | Extended Theoretical and Empirical Justification |
|---|---|---|---|
| **Motivation** | How worried are you about COVID-19's impact? (COV_Wor) | Very worried (5) to Not worried at all (1) | Worry and fear are core components of reflective and automatic motivation in COM-B, capturing emotional drivers that shape intention formation. Studies shows that emotional responses linked to perceived threat—such as functional fear—are associated with stronger adherence to preventive behaviours during COVID-19 [10]. This item therefore reflects emotional–evaluative motivation. |
| **Behaviour** | Have you self-isolated in the past 7 days? (COV_SelfIso) | Yes (1), No (0) | Self-isolation behaviour is the focal observable outcome. Operationalising behaviour as a binary indicator aligns with the COM-B requirement for a clear, measurable behavioural endpoint. This item directly captures the action of interest. |

**Table A1**: Extended theoretical and empirical justification for mapping ONS survey items onto COM-B constructs.

Item-to-construct mapping followed an iterative expert review process as shown in Table A1. Three researchers with experience in behavioural science and COM-B independently reviewed all ONS items and proposed initial classifications based on theoretical definitions from Michie et al. (2011) [2]. Discrepancies were discussed in two consensus meetings, where final assignments were agreed using explicit criteria: conceptual alignment with COM-B domains, relevance to self-isolation behaviour, and consistency with prior empirical applications of the framework.

# Appendix B Average variance extracted (AVE)

| Construct | AVE | Interpretation |
| --- | --- | --- |
| Capability | 0.126 | Reflects heterogeneous indicators approximating capability using secondary data not designed for COM-B measurement |
| Opportunity | 0.353 | Indicates moderate convergent validity driven primarily by social opportunity indicators |
| Motivation | 0.208 | Reflects mixed reflective and emotional indicators capturing self-oriented motivation |

**Table B2**: Average Variance Extracted (AVE) values for COM-B constructs. AVE values are reported for transparency and should be interpreted in the context of secondary data analysis using indicators not originally designed for psychometric scale development.

Consistent with the use of secondary data and heterogeneous indicators, AVE values were modest (Capability = 0.126; Opportunity = 0.353; Motivation = 0.208) and should be interpreted as reflecting data constraints rather than theoretical misalignment with the COM-B framework (see Table B2).

# References

[1] Killgore, W.D., Cloonan, S.A., Taylor, E.C., Dailey, N.S.: Loneliness: A signature mental health concern in the era of covid-19. Psychiatry Research **290**, 113117 (2020) https://doi.org/10.1016/j.psychres.2020.113117

[2] Michie, S., Van Stralen, M.M., West, R.: The behaviour change wheel: A new method for characterising and designing behaviour change interventions. Implementation Science **6**(1), 42 (2011) https://doi.org/10.1186/1748-5908-6-42

[3] Williams, S., Dienes, K., Jaheed, J., Wardman, J.K., Petts, J.: Effectiveness of communications in enhancing adherence to public health behavioural interventions: a covid-19 evidence review. Philosophical Transactions of the Royal Society A **381**(2257) (2023) https://doi.org/10.1098/rsta.2023.0129

[4] Benham, J.L., Lang, R., Kovacs Burns, K., MacKean, G., Léveillé, T., McCormack, B., *et al.*: Attitudes, current behaviours and barriers to public-health

measures that reduce covid-19 transmission: A qualitative study to inform public-health messaging. PLoS ONE **16**(2), 0246941 (2021) https://doi.org/10.1371/journal.pone.0246941

[5] Christakis, N.A., Fowler, J.H.: Social contagion theory: examining dynamic social networks and human behavior. Statistics in Medicine **32**(4), 556–577 (2013) https://doi.org/10.1002/sim.5408

[6] Indrayathi, P.A., Januraga, P.P., Pradnyani, P.E., Gesesew, H.A., Ward, P.R.: Perceived social norms as determinants of adherence to public health measures related to covid-19 in bali, indonesia. Frontiers in Public Health **9**, 646764 (2021) https://doi.org/10.3389/fpubh.2021.646764

[7] Tunçgenç, B., El Zein, M., Sulik, J., Newson, M., Zhao, Y., Dezecache, G., Deroy, O.: Social influence matters: We follow pandemic guidelines most when our close circle does. British Journal of Psychology **112**(1), 235–256 (2021) https://doi.org/10.1111/bjop.12491

[8] Smith, L.E., Potts, H., Amlot, R., Fear, N.T., Michie, S., Rubin, G.J.: Adherence to protective behaviours during the covid-19 pandemic: a cross-sectional study of vulnerable adults in the uk. BMJ **370**, 2911 (2020) https://doi.org/10.1136/bmj.m2911

[9] Wright, L., Steptoe, A., Fancourt, D.: Health risk factors and protective behaviours during covid-19: a longitudinal analysis. Scientific Reports **11**(1), 9028 (2021) https://doi.org/10.1038/s41598-021-88314-4

[10] Harper, C.A., Satchell, L.P., Fido, D., Latzman, R.D.: Functional fear predicts public health compliance in the covid-19 pandemic. International Journal of Mental Health and Addiction (2020) https://doi.org/10.1007/s11469-020-00281-5
